# 15 years of genome-wide association studies and no signs of slowing down

**DOI:** 10.1038/s41467-020-19653-5

**Published:** 2020-11-19

**Authors:** Ruth J. F. Loos

**Affiliations:** 1 The Charles Bronfman Institute for Personalized Medicine, Icahn School of Medicine at Mount Sinai, New York, NY USA; 2 The Mindich Child Health and Development Institute, Icahn School of Medicine at Mount Sinai, New York, NY USA

**Keywords:** Genome-wide association studies, Diseases

## Abstract

Over the past 15 years, genome-wide association studies (GWASs) have generated a wealth of new information. Larger samples sizes, refined phenotypes and higher-resolution genome-screens will continue to drive gene discovery in years ahead. Meanwhile, GWAS loci are increasingly translated into new biology and opportunities for clinical care.

When the Human Genome Project (HGP) was launched in 1993, the expectation was that genomics would transform clinical care, providing the insights needed to develop better diagnostic, prognostic, preventive, and therapeutic strategies for rare and common diseases. Upon completion of the HGP in 2003, the genome-wide association approach was hailed as the key gene discovery paradigm to translate these expectations into practice. Genome-wide association studies (GWASs) screen the genome for associations between millions of genetic variants and a disease or trait without any a priori hypothesis. As such, GWASs may reveal new genes and pathways not previously implicated in the disease pathology.

While the very first GWAS was published in 2005^1^, it was the Wellcome Trust Case Control Consortium (WTCCC) that in 2007 set the stage for many more GWASs to come^2^. With their pivotal paper, the WTCCC demonstrated that combining forces (large sample sizes), a rigorous study design (discovery and replication stages), and stringent criteria (multiple testing corrected significance level) were needed for reproducible discoveries. By the time Nature Communications was launched in April 2010, GWASs had already accelerated the rate of gene discovery to an unprecedented scale, identifying more than 3000 unique loci for over 250 disease/trait outcomes^3^. These numbers increased exponentially over the subsequent 10 years, and to date, more than 4300 papers have reported on 4500 GWASs and over 55,000 unique loci for nearly 5000 diseases and traits^3^. Summary statistics for most GWASs have been made publicly available^3^, and a number of user-friendly data portals allow scientists to query GWAS data freely (Box 1).

## Box 1

Selection of databases and browsers

- **Genome-wide association studies**
  **GWAS catalog** https://www.ebi.ac.uk/gwas/
  The GWAS catalog is a searchable database of SNP-trait associations for published GWAS.
  **Knowledge Portal Network** http://www.kp4cd.org/
  The Knowledge Portal Network is a software platform that integrate, interpret, and present human genetic and genomic data to spark insights into complex diseases.
  **PhenoScanner** http://www.phenoscanner.medschl.cam.ac.uk/
  PhenoScanner is a curated database holding publicly available results from large-scale genome-wide association studies.
- **Multi-omics**
  **GTex (Genotype-Tissue Expression) eQTL Browser** https://gtexportal.org/home/
  GTex is a resource to study human gene expression and regulation and its relationship to genetic variation.
  **ENCODE: Encyclopedia of DNA Elements** https://www.encodeproject.org/
  *ENCODE is a comprehensive list of functional elements in the human genome, including elements that act at the protein and RNA levels, and regulatory elements that control cells and circumstances in which a gene is active*.
  **Roadmap Epigenomics Mapping Consortium** http://www.roadmapepigenomics.org/
  Roadmap Epigenomics Mapping Consortium built a resource of human epigenomic data to catalyze basic biology and disease-oriented research.
- **Mendelian Randomization analyses**
  **MR base** http://app.mrbase.rg
  MR-Base automates implementation of two-sample Mendelian randomization, including effect allele harmonization across separate studies, LD pruning to ensure independence of genetic variants and diagnostic and sensitivity analyses.
- **Polygenic scores**
  **Polygenic score catalog** https://www.pgscatalog.org
  The PGS Catalog is an open database of published polygenic scores (PGS). Each PGS has been annotated with relevant metadata.
  **PRS Atlas** http://mrcieu.mrsoftware.org/PRS_atlas/
  The PRS Atlas is a web application to query findings from an analysis of 162 polygenic risk scores and 551 complex traits using data from the UK Biobank study.
- **Diversity in genome-wide association studies**
  **GWAS Diversity Monitor** https://gwasdiversitymonitor.com/
  The GWAS Diversity Monitor monitors the diversity of participants across all published GWAS.

This steep increase has been driven by continuous advancements in the field. First, the decreasing cost of genome-wide genotyping arrays, now >20 times less expensive than 15 years ago, has allowed more studies to participate in gene discovery efforts. Recent GWAS meta-analyses for traits such as kidney function (eGFR), blood pressure, and insomnia, have already exceeded 1 million participants. Second, the number of variants tested has increased 20-fold; from ~500,000 variants in the early days to nearly 10 million variants in the latest GWASs. In particular, imputation of untyped/missing variants, combined with updated reference panels of genetic variation (e.g. the International HapMap, the 1000 Genomes Project, Haplotype Reference Consortium) and improved genome-wide genotyping arrays have substantially increased the number of variants tested, not only of common, but also of rare(r) protein-coding and structural variants. Third, while in the beginning, GWASs focused mostly on common risk factors and disease outcomes, increasingly, new and more refined phenotypes are being studied, such as imaging-derived traits, response to interventions or medications, and multi-omics outcomes. Fourth, advanced statistical analyses and sophisticated modeling have been key in securing continued progress in gene discovery; e.g. multivariate GWASs to identify loci that affect multiple traits/diseases simultaneously, cluster analyses across intermediate traits to deconstruct the heterogeneity of disease, genome-wide gene-environment interaction analyses to identify loci of which the association is sensitive to environmental factors, and gene-burden and pathway-based GWASs that combine genetic variants in biological meaningful groups.

While there are no signs that gene discovery is slowing down, identifying genetic associations is only the first step of a long journey^4^. Over the years, follow-up analyses of GWAS loci have become an integral part of GWAS reports. These analyses fall into two broad categories of research: the first category focuses on translation of genetic loci into new biological insights, while the second category of studies aims to implement this new knowledge in clinical care.

The translation of genetic loci into biological mechanisms that underlie disease has been one of the most arduous tasks. A major challenge is the exploration of the functional consequences of identified variants, as the vast majority (~90%) of GWAS-identified variants lie in the non-coding parts of the genome. Increasingly, multi-omics data across multiple cell types and tissues are being generated at a genome-scale (Box 1). Numerous computational pipelines are being developed that integrate these multi-omics data with genome-wide association data to determine the regulatory impact of a locus, to prioritize the likely causal variant and/or gene and to determine the tissues that are key to the pathogenesis of the disease^5^. For example, using a series of computational tools, more than 20% of loci associated with type 2 diabetes (T2D) have been mapped to the most likely causal variant^6^. Subsequent validation, using targeted molecular experiments, is critical to further establish the role of the prioritized genes and/or variants. For example, in vitro functional analyses in adipocytes and adipocyte progenitors, combined with in vivo adipose phenotyping of mutant zebrafish, confirmed *RSPO* as the likely causal gene in a GWAS locus for fat distribution, influencing peripheral fat storage^7^. Ultimately, prioritized genes need to be validated in human models, as demonstrated for a mutation in *SLC30A8* (encoding ZnT8). In a recall-by-genotype study with detailed metabolic phenotyping, it was shown that carriers were protected from T2D through enhanced glucose responsiveness and proinsulin conversion, making *SLC30A8*/ZnT8 an appealing target for antidiabetic therapies^8^. The generation of new data and development of advanced technologies and analytical approaches will continue to facilitate the translation of a growing number of GWAS loci into meaningful biology and clinical targets in many years ahead.

Besides translational research, GWASs have generated an enormous amount of information that has fueled applied epidemiological research. Currently, the most prominent applications are Mendelian Randomization (MR) and polygenic scores. MR is used to determine causality between an exposure (e.g. health-related behaviors, biomarker) and an outcome (e.g. disease). Genetic variants that are robustly associated with the exposure are used to randomize a population in those with high exposure (i.e. carriers of the risk alleles) and those with low exposure (i.e. carriers of non-risk alleles). If the same genetic variants also associate with the disease outcome through their association with the exposure, causality between exposure and disease can be inferred. MR analyses have been performed to confirm (or refute) causal relationships between numerous correlated traits and diseases. In recent years, this approach is also being used to validate putative drug targets prior to the initiation of clinical trials, as well as to determine potential side effects of therapeutic interventions^9,10^. Web-based analysis platforms (Box 1), using publicly available data, allow researchers to perform MR analyses for their chosen exposures and outcomes^11^.

The use of polygenic risk scores (PRSs) in disease risk stratification and precision medicine is another popular application of GWAS data. A PRS estimates an individual’s lifetime genetic susceptibility to disease by aggregating the effects conferred by the millions of variants tested in a GWAS. The assumption is that individuals with a very high PRS have an above-average lifetime genetic risk of developing a given disease. However, as many common diseases are determined by genetic and non-genetic factors, the clinical utility of a PRS needs to be assessed in the context of existing clinical predictors of risk^12^. It has been postulated that knowing an individual’s PRS for a given disease, may help health care providers with decisions on their patient’s participation in screening programs, lifestyle modifications, and/or preventive treatments. While implementation of PRSs in routine clinical care has a long road ahead, the most promising evidence for its utility has been seen in cardiovascular diseases and cancer^13,14^. PRSs for published GWASs have been made publicly available (Box 1).

Despite tremendous progress, GWASs have been met with criticism. Some researchers have argued that because GWAS loci confer a small increase in disease risk and explain only a fraction of the heritability, their contribution to disease cannot be that important. However, as more loci are being translated into biological insights, there is growing evidence that the strength of association of a GWAS locus is not proportional to its biological importance. As in-depth mapping of GWAS loci requires a multi-disciplinary team of scientists to integrate a wide range of expertise and data, the gap between geneticists and non-geneticists is slowly closing. Another important shortcoming is the continued underrepresentation of individuals of non-European ancestry. Large-scale GWAS efforts have disproportionally focused on European ancestry populations with only ~10% of all GWAS participants being of non-European descent (Box 1)^15^. Lack of representation of diverse populations not only limits the transferability of GWAS results across populations, but may result in inequitable access to clinical care informed by genetic research. More initiatives such as the PAGE Study, H3Africa, the African Genome Variation Project and GenomeAsia 100k are needed to reverse this Euro-centricity.

Despite its critics, current signs suggest that GWASs will be around for much longer. Sample sizes are expected to increase even more rapidly than before—easily exceeding 5 million participants—as data from large-scale biobanks and cohorts, such as the UK Biobank, the Million Veterans Project, All of Us, and 23andme, become available. This will not only result in additional GWAS loci, including those driven by rarer and/or population-specific variants, but also in more precise per-variant effect estimates, which is crucial to improve the predictive ability and clinical utility of future PRSs. The increasing availability of GWAS data from non-European populations will further maximize gene discovery and reduce health disparities. Furthermore, imputation of the latest reference panels, based on data from whole-genome sequencing (WGS) projects (e.g. TOPMed Program, UK Biobank), will soon allow testing the association of more than 150 million variants, providing a much more affordable alternative to WGS. In addition, the increasing availability of high-throughput genome-scale technologies for mapping sites of regulatory impact will accelerate the translation of GWAS loci into new biological insights. These developments, together with continued technological and analytical advances, will keep driving innovation in the GWAS field for years to come.

Over the past 15 years—while the low-hanging fruits were being picked—the field has matured tremendously, such that today we have the cutting-edge technologies, sophisticated analytical tools, and comprehensive multi-omics databases to begin to decipher the complex underlying biology of GWAS loci and their role in health and disease. Clearly, GWASs show no signs of slowing down.
